# Genome-Wide Identification and Expression Profile Analysis of the NF-Y Transcription Factor Gene Family in *Petunia hybrida*

**DOI:** 10.3390/plants9030336

**Published:** 2020-03-06

**Authors:** Qian Wei, Shiyun Wen, Chuying Lan, Yixun Yu, Guoju Chen

**Affiliations:** 1 Guangdong Key Laboratory for Innovative Development and Utilization of Forest Plant Germplasm, College of Forestry and Landscape Architecture, South China Agricultural University, Guangzhou 510642, China; weiqian@scau.edu.cn (Q.W.); sywen@stu.scau.edu.cn (S.W.); cylan@stu.scau.edu.cn (C.L.); yuyixun@scau.edu.cn (Y.Y.); 2 College of Horticulture, South China Agricultural University, Guangzhou 510642, China

**Keywords:** *Petunia hybrida*, *NF-Y* gene family, gene expression, abiotic stress

## Abstract

Nuclear Factor Ys (NF-Ys) are a class of heterotrimeric transcription factors that play key roles in many biological processes, such as abiotic stress responses, flowering time, and root development. The petunia (*Petunia hybrida*) is a model ornamental plant, and its draft genome has been published. However, no details regarding the NF-Y gene family in petunias are available. Here, 27 NF-Y members from the petunia genome were identified, including 10 PhNF-YAs, 13 PhNF-YBs, and 4 PhNF-YCs. Multiple alignments showed that all PhNF-Y proteins had clear conserved core regions flanked by non-conserved sequences. Phylogenetic analyses identified five pairs of orthologues NF-YB proteins from *Petunia* and *Arabidopsis*, and six pairs of paralogues NF-Y proteins in *Petunia*. Analysis of the gene structure and conserved motifs further confirmed the closer relationship in each subfamily. Bioinformatics analysis revealed that 16 PhNF-Ys could be targeted by 18 miRNA families. RNA-seq results showed that expression patterns of PhNF-Ys among four major organs (leaf, stem, flower, and root) were clustered into six major groups. The stress response pattern of PhNF-Ys was identified under cold, heat, drought, and salinity treatments. Based on the RNA-seq data, we found that 3 genes responded to drought, 4 genes responded to salt, 10 genes responded to cold, and 9 genes responded to hot. In conclusion, this study provides useful information for further studying the functions of *NF-Ys* in stress response.

## 1. Introduction

Abiotic stress, such as temperature, salinity, and drought, can cause great damage to plant growth and development. To survive, plants have evolved strategies to tolerate stress, including developmental, physiological, morphological, ecological, biochemical, and molecular mechanisms [1]. Members of many transcription factor (TF) families, such as AP2/ERF, MYB/MYC, zinc-finger protein, and NAC, play important roles at molecular levels by increasing or decreasing the expression of stress responsive genes [2]. Compared to those TF families, Nuclear Factor Y (NF-Y) is a relatively late reported TF family that participates in abiotic stress response.

NF-Y is a heterotrimeric complex with three distinct subunits, NF-YA, NF-YB, and NF-YC, that binds to the CCAAT element in the promoters of genes [3]. All three subunits contain the conserved DNA binding domains and subunit interaction domains [4]. To form heterotrimeric complexes, NF-YB and NF-YC, who possess H2B and H2A motifs, respectively, form a tight histone dimer in the cytoplasm, and the dimer then translocates into the nucleus where it can interact with NF-YA to form the final heterotrimer [5]. Since NF-YA can specifically bind to the CCAAT boxes, the heterotrimer acts as a transcript factor to regulate downstream genes containing CCAAT binding site in promoter [5].

NF-Y is highly conserved in all higher eukaryotes [3]. In yeast and animals, each NF-Y subunit is encoded by a single gene, but in plants, NF-Y subunits are multigene families, which implies that the heterotrimeric NF-Y complexes may act in diverse combinations of subunits for the specific transcriptional regulation [6]. Although no complete NF-Y complex has been reported in plants, the functions of individual subunits are becoming clearer. Many studies have shown that single NF-Ys play important roles in many processes, such as chloroplast biogenesis [7], photomorphogenesis [8], embryogenesis [9], nodule development [10], root growth [11], seed development [12], flowering time [13], and stress response [14,15,16,17,18,19,20].

For abiotic stress response, different NF-Y members play positive or negative roles via different mechanisms. Overexpression of *AtNF-YA1* or *TaNF-YA10-1* significantly increased the plant’s sensitivity to salinity [16,21], while overexpression of *SiNF-YA5* or *OsHAP2E* could enhance salt tolerance [14,22]. *GmNF-YA3* positively modulated drought tolerance of *Arabidopsis* through the ABA-dependent pathway, while *OsNF-YA7* conferred drought tolerance of rice in an ABA-independent manner [23,24]. Previous studies revealed that many *NF-YA* genes were involved in the miR169-regulated abiotic stress response [25,26]. Similar to the case of the NF-YA subfamily, *AtNF-YB1* and its homologues *ZmNF-YB2* enhanced drought tolerance of *Arabidopsis* and rice, respectively, neither in an ABA-dependent manner nor in an ABA-independent manner [17], but *PdNF-YB7* increased drought tolerance through upregulating downstream genes of the ABA-dependent pathway in *Arabidopsis* [27]. Moreover, the individual *NF-YB* gene was reported to be involved in multiple stresses. *SiNF-YB8* can enhance drought and osmotic tolerance in tobacco [28]. Overexpression of *PwNF-YB3* can significantly improve tolerance of seedlings under salinity, drought, and osmotic stress [18]. *NF-YC* also plays important functions in response to different kinds of stresses. *CdtNF-YC1* increased drought and salinity tolerance in rice through regulating genes in both ABA-dependent and ABA-independent pathways [29]. *AtHAP5A* improved freezing stress resistance in *Arabidopsis* independent of the CBF pathway [30].

To date, NF-Y transcription factors have been identified in several species, including 33 *NF-Y* genes in *Arabidopsis thaliana* [31], 37 *NF-Y* genes in *Triticum aestivum* [32], 28 *NF-Y* genes in *Oryza sativa* [33], 68 *NF-Y* genes in *Glycine max* [34], 33 *NF-Y* genes in *Brassica napus* [35], 36 *NF-Y* genes in *Brachypodium distachyon* [36], 34 *NF-Y* genes in *Vitis vinifera* L. [37], 19 *NF-Y* genes in *Citrullus lanatus* [38], 33 *NF-Y* genes in *Sorghum bicolor* (L.) Moench [8], 22 *NF-Y* genes in *Citrus sinensis* [39], 33 *NF-Y* family genes in *Phyllostachys edulis* [40], 39 *NF-Y* genes in *Setaria italica* [28]. However, no information is available on the *NF-Y* gene family in petunias (*Petunia hybrida*).

The petunia (*Petunia hybrida*) is a widely used landscaping plant around the world. Adverse environmental conditions greatly limit its display period and ornamental value. The petunia is also a model plant for studying ornamental plants. Taking advantage of the current draft of the petunia genome, and based on the significance of the NF-Y family in plant development and stress response, we attempted to characterize the petunia *NF-Y* gene family. In this study, we identified 27 *NF-Y* genes from the petunia genome, analyzed phylogenetic relationships, and conserved protein motifs, exon-intron structures, and micro RNA (miRNA) targeting of the *NF-Y* genes to further characterize PhNF-Ys. In addition, we analyzed *NF-Y* gene expression profiles in various tissues and in response to abiotic stresses, including cold, heat, drought, and salt. Our results should provide insights into the function of the NF-Y gene family in petunias.

## 2. Results

### 2.1. Identification of the NF-Y Family Members

A total of 27 NF-Y proteins of Petunia (Table 1) were identified through BLASTP by using full-length amino acid sequences of 36 AtNF-Y proteins. The identified NF-Y proteins were named PhNF-YA1-PhNF-YA10, PhNF-YB1-PhNF-YB13, and PhNF-YC1-PhNF-YC4. The characteristics of the PhNF-Y sequences are listed in Table 1. The lengths of gene sequences ranged from 327 bp to 1065 bp. The lengths, the molecular weights (MWs), and the isoelectric point (pI) values of these proteins ranged from 108 aa to 327 aa, from 11.92 kDa to 39.76 kDa, and from 4.25 to 10.39, respectively.

### 2.2. Multiple Alignment and Conserved Motifs of NF-Y Proteins

**Figure 1:**
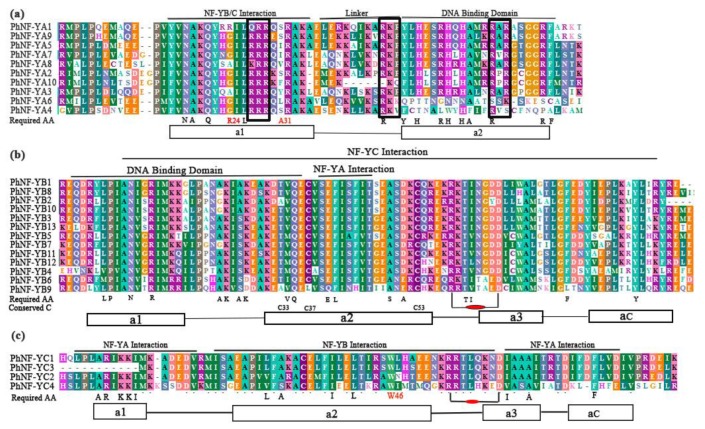
Multiple alignments of conserved domains in three subfamilies of PhNF-Ys. The DNA binding and subunit interaction domain are represented. The alpha-helices (**rectangles**) and coils (**black lines**) are marked on the bottom. Required amino acids (Required AA) are given on the bottom of the alignment. (**a**) Multiple alignments of conserved domains in the PhNF-YA family. The three black boxes in the A group are the residual clusters required for nuclear targeting. (**b**) Multiple alignments of conserved domains in the PhNF-YB family. Conserved cysteines C33, C37, and C53 of PhNF-YB are marked. (**c**) Multiple alignments of conserved domains in the PhNF-YC family. The putative arginine (R)-aspartate (D) pairs in (**b**) and (**c**) are marked.

Multiple alignments showed that all PhNF-Y proteins had clear conserved core regions flanked by non-conserved sequences. The core regions of most petunia NF-YA proteins contained two conserved domains, one for the NF-YB/C interaction (20AA) and the other for binding CCAAT domain in the promoter (21AA) (Figure 1a). The two domains were separated by a conserved linker (11AA). However, the PhNF-YA6 and PhNF-YA4 lacked the conserved DNA binding domain of the NF-YA subfamily, which was necessary for the recognition of the CCAAT domain. In addition, a conserved 12 AA sequence with an unknown function was identified in front of the NF-YB/C interaction domain. We also noticed that most amino acid residues required in mammals and yeast [3,41] were remained in most petunia NF-YA proteins. For these required amino acid residues, the arginine (R24) was often replaced by alanine or glycine, and the alanine (A31) was mutative in plant NF-YA proteins [31]. In addition, eight of the petunia NF-YA proteins contained three predicted nuclear localization signals [5,42] (black boxes in Figure 1a).

The conserved region of *Petunia* NF-YB proteins had structural similarities with H2B histone. This region possessed characteristic domains for DNA-binding and NF-YA/C interaction (Figure 1b). The required amino acid residues were highly conserved in PhNF-YB proteins, particularly the functionally important protein residues. The characteristic intra-chain arginine-aspartate (R58-D65) bidentate pairs, which were necessary in stabilizing the NF-YB/C heterodimer, were absolutely conserved [43]. Notably, similarly with *Arabidopsis* and soybean, the disulfide bond between C33 and C37 was not presented, which played a crucial role in the translocation of NF-YB/C into nuclei in humans and *Aspergillus nidulans* [43]. This evidence further confirmed the conserved histone structure and changed the model of nucleus translocation of NF-YB proteins in plants.

The PhNF-YC proteins contained highly conserved NF-YA/B interaction domains and had structural similarities with H2A histone [43]. The required amino acids were conserved with few alterations by similar functional residues [44] (Figure 1c). Similarly with NF-YB subunits, the arginine (R54) and aspartate (D61) pairs were present in all petunia NF-YCs [43]. Another specific feature-tryptophan (W46) at the end of α2, which made the specific interaction of NF-YC and NF-YB, was also absolutely conserved in all PhNF-YCs.

**Figure 2:**
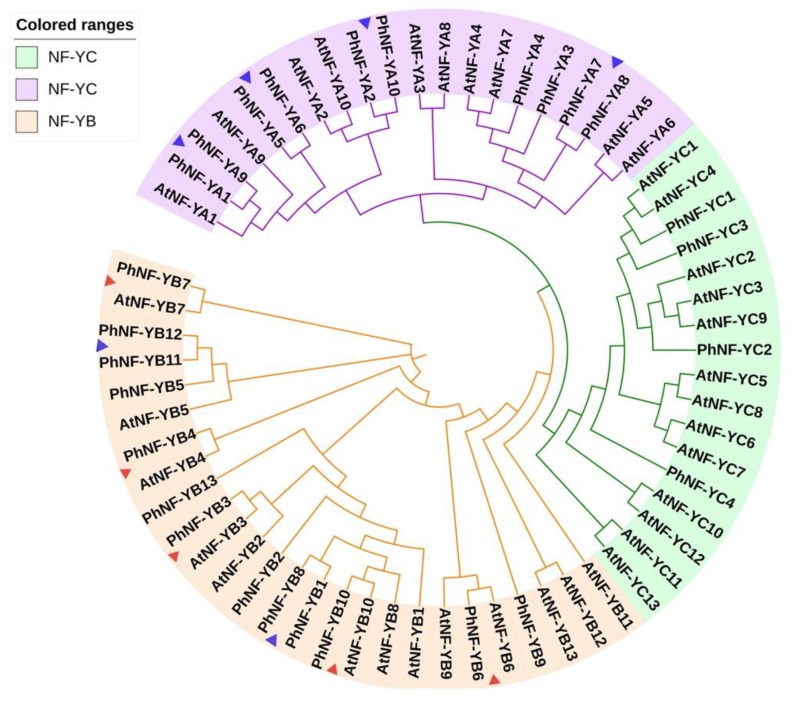
Phylogenetic analysis of NF-Y proteins in *Arabidopsis thaliana* and *Petunia hybrida*. Three subfamilies are represented by different colors. ▲ indicates five pairs of orthologues; ▲ indicates six pairs of paralogues.

To reveal the evolutionary relationship and potential function of PhNF-Ys, an unrooted neighbor-joining phylogenetic tree was constructed using 63 full-length protein sequences of NF-Ys from *Petunia* and *Arabidopsis* (Figure 2). The phylogenetic analysis revealed that the 63 NF-Y proteins were clustered into three groups: NF-YA (purple), NF-YB (orange), and NF-YC (blue), which were consistent with our subfamily classifications of the PhNF-Ys (Table 1). Based on the genetic relations and the reported functions of AtNF-Ys, the functions of PhNF-Y members could be concluded and further tested. Notably, five pairs of NF-YB orthologues (AtNF-YB7 and PhNF-YB7; AtNF-YB4 and PhNF-YB4; AtNF-YB10 and PhNF-YB10; AtNF-YB6 and PhNF-YB6; AtNF-YB3 and PhNF-YB3) were found in the NF-YB group, suggesting a significant possibility of the similar biological functions between the NF-YB orthologues. Moreover, six pairs of paralogues were identified: PhNF-YB1 and PhNF-YB8; PhNF-YB11 and PhNF-YB12; PhNF-YA1 and PhNF-YA9; PhNF-YA5 and PhNF-YA6; PhNF-YA2 and PhNF-YA10; PhNF-YA7 and PhNF-YA8.

### 2.3. Gene Structures and Conserved Motifs of PhNF-Y Members

**Figure 3:**
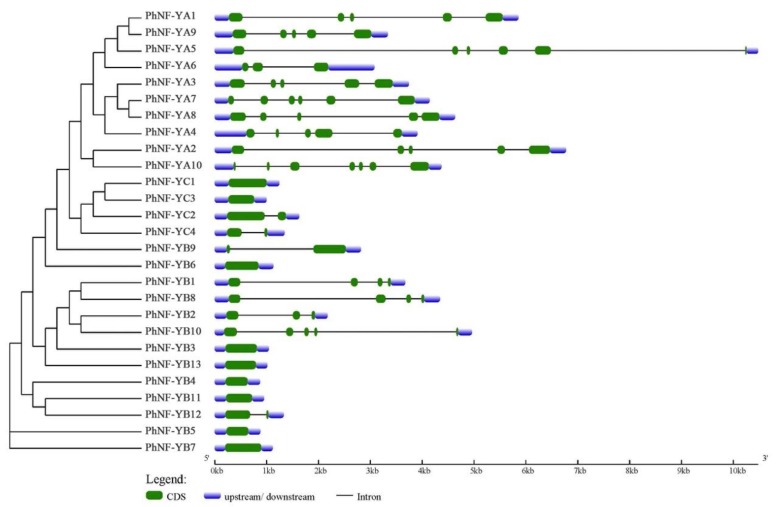
The exon-intron structure of the NF-Y gene family in petunias.

To further understand the evolutionary relationships in the petunia NF-Y family, the exon-intron structures were analyzed using the GSDS website (Figure 3; http://gsds.cbi.pku.edu.cn/index.php). Most of the PhNF-YAs had five or six exons with similar distribution, an exception being PhNF-YA6. Half of the PhNF-YBs had no intron, and the other half were variable, but the members involved in the same clade had similar numbers of exons and introns. For the PhNF-YC subfamily, PhNF-YC1 and PhNF-YC3 had only one exon, and PhNF-YC2 and PhNF-YC4 had two exons. The gene structures of the NF-Y members were positively associated with their phylogenetic relationship and were consistent with the previous reports [8,43].

**Figure 4:**
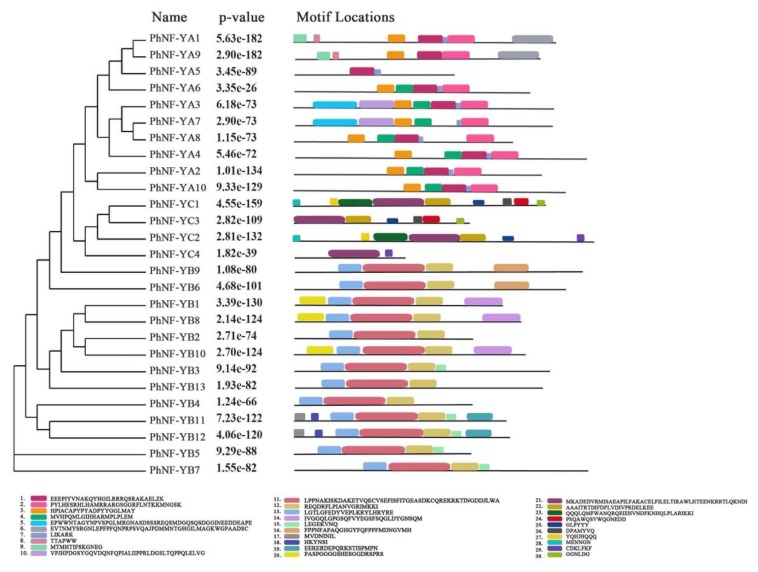
The motif structure of the NF-Y gene family in petunias. Ten conserved motifs of each subfamily were displayed in different colors. The motif sequence information was provided at the bottom.

The putative conserved motifs of 27 PhNF-Ys were identified using Multiple Em for Motif Elicitation (Figure 4; MEME 5.1.0). Ten conserved motifs were recognized in each subfamily, and the lengths of the motifs ranged from 6 to 50 amino acids. For the PhNF-YA subfamily members, Motif 1, 2, and 3 were presented in 8 of the 10 NF-YAs. PhNF-YA4 lacked Motif 1 and 3, and NF-YA8 lacked Motif 3. Furthermore, the members of the same clade contained similar motif numbers and distribution patterns, indicating that they might have similar functions. All PhNF-YB proteins contained Motif 11, 12, and 13, and the variation of motifs were consistent with the phylogenetic relationships. In the case of the PhNF-YC subfamily, Motif 21, 22, and 23 were observed in all four members, and Motif 25, 26, 27, and 28 were found in PhNF-YC2 and PhNF-YC4.

### 2.4. miRNA Target Site Prediction and Validation

It is well known that microRNAs can regulate target genes though cleavage of mRNA or translation inhibition [45]. In the petunias, 58 mature miRNA sequences that belonged to 39 miRNA families have been reported. Twenty-five were conserved at a wider taxonomic range, 19 were specific for the Solanaceae, and 13 variants were unique to the petunia [46].

**Figure 5:**
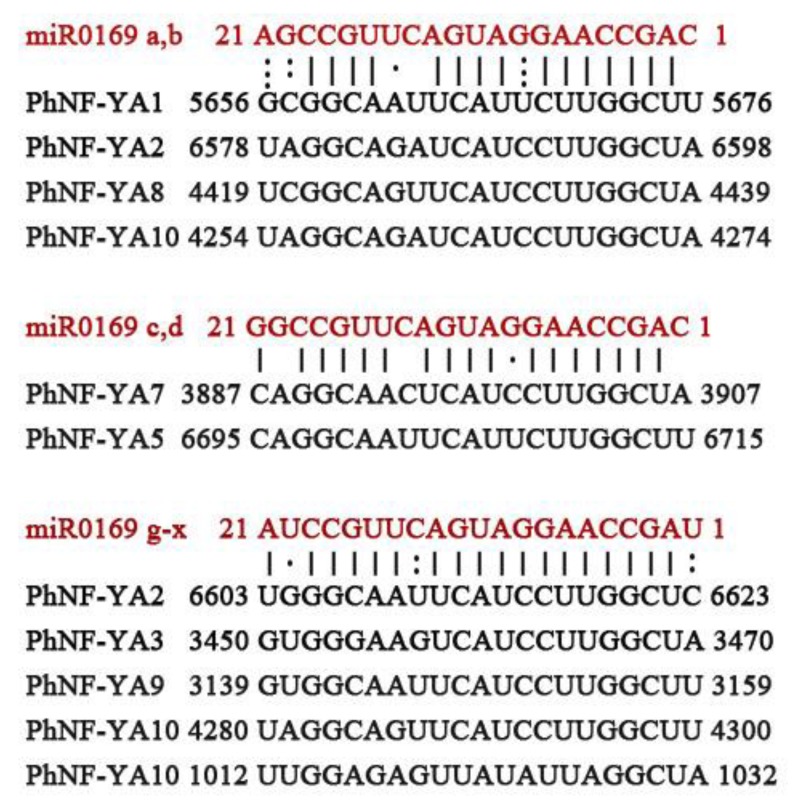
Nuclear acid sequence alignments of mature Ph-miR169 and Ph-miR169 target sites in PhNF-YA family members.

All these sequences were used to predict target transcript candidates of PhNF-Ys. The results showed that 16 PhNF-Ys could be regulated by 18 miRNA families (Table S1). Eight PhNF-YAs, 6 PhNF-YBs, and 2 PhNF-YCs had target sites of 14, 5, and 3 miRNA families, respectively. In most cases, one miRNA family had one or two target genes in the NF-Y family. However, eight NF-YA members had target sites of miRNA169 (Figure 5), which was consistent with previous reports [47,48]. These results indicated that the miR169-NF-YA module existed in *Petunia* and that PhNF-YAs had more uncovered relationships with other miRNA families.

### 2.5. Expression Profiles of the PhNF-Ys in Different Tissues

**Figure 6:**
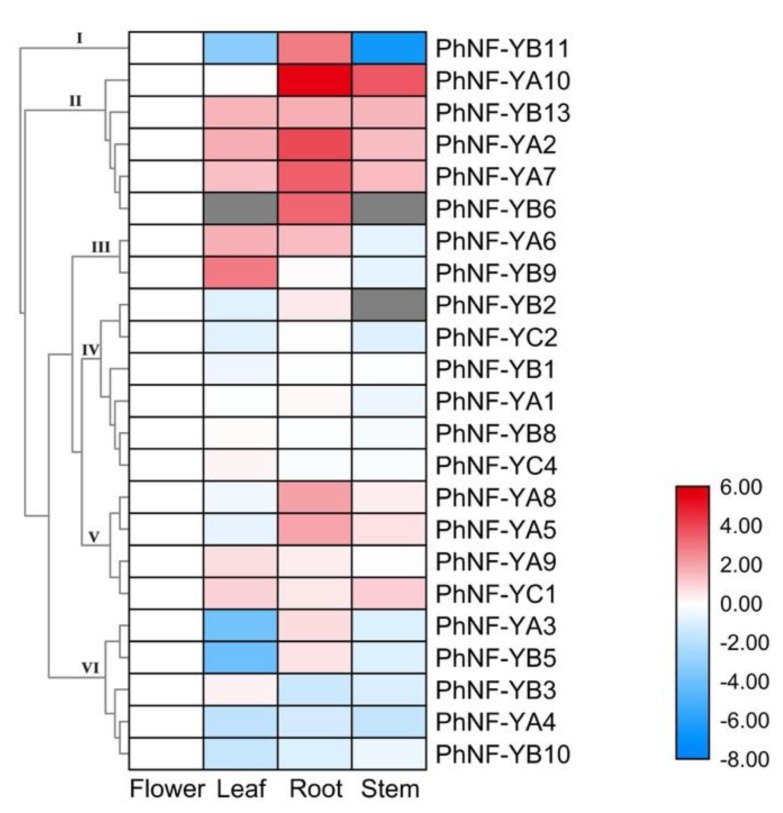
Expression profiles of PhNF-Ys in different tissues. A cluster dendrogram is shown on the left. NF-Ys are divided into six major groups based on their expression patterns. Grey boxes indicate that transcription was not detected at that timepoint. The bar on the lower right corner represents log_2_-based expression values transformed from fragments per kilobase of exon per million reads mapped (FPKM) values. Blue and red indicate decreased and increased transcript abundance, respectively, compared to controls (**flower**).

To investigate the biological functions of *PhNF-Y genes* in the petunias, the expression patterns of *PhNF-Y* genes were analyzed in four major tissues (leaf, stem, root, and flower) of plants under normal conditions. Based on the RNA-seq data, the corresponding expression data of 23 *PhNF-Y* genes were collected, and the other four *NF-Y* genes were not detected and were thus omitted from the analysis. As shown in Figure 6, 23 *PhNF-Y* genes were detected and clustered into six subsets based on their expression patterns. *PhNF-Ys* in Subset I and II showed the highest transcript abundance in the root. *PhNF-Ys* in Subset III showed relatively higher expression in leaf (*PhNF-YA6* and *PhNF-YB9*) and root (*PhNF-YA6*). The expression of *PhNF-Ys* in Subset IV was not obviously different among different tissues. The expression of *PhNF-Ys* in Subset V presented a moderate change among tissues. *PhNF-Ys* in Subset VI presented relatively higher transcript abundance in the flower. The different expression patterns suggested diverse functions of *PhNF-Ys* during petunia growth and development.

### 2.6. Expression Profiles of the PhNF-Ys under Abiotic Stress

**Figure 7:**
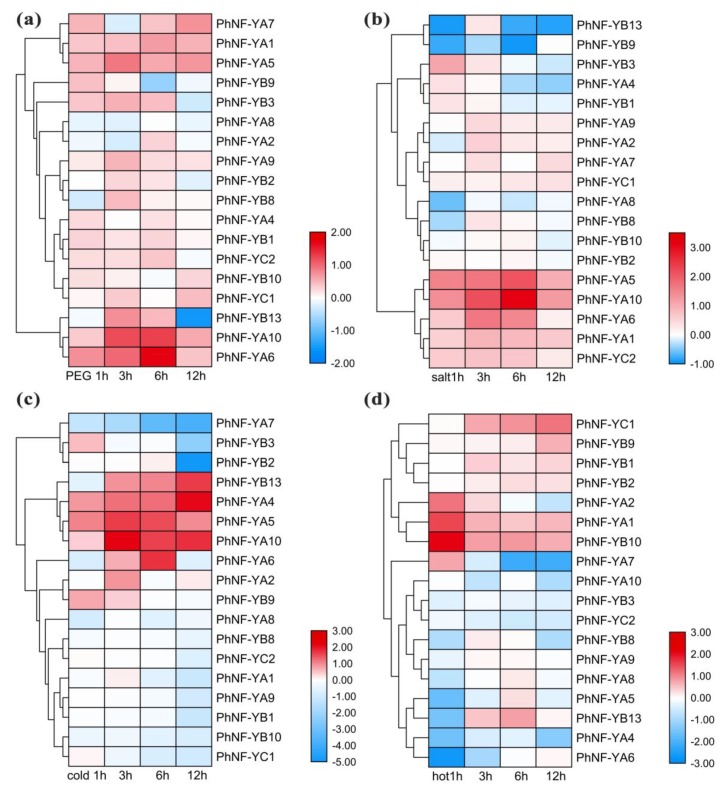
Expression profiles of *PhNF*-*Ys* in response to abiotic stress treatments. (**a**) Expression profiles of *PhNF*-*Ys* under drought treatment (20% PEG). (**b**) Expression profiles of *PhNF*-*Ys* under salt treatment (500 mM). (**c**) Expression profiles of *PhNF*-*Ys* under cold treatment (4 °C). (**d**) Expression profiles of *PhNF-Ys* under hot treatment (40 °C). The treatments and timepoint are marked at the bottom of each column. The bars on the right bottom indicate fold-change values (log_2_ values) with red representing increased transcript abundance and blue indicating decreased transcript abundance.

Previous reports have verified that NF-Y members could respond to various abiotic stresses [49]. We performed RNA-seq to identify the expression levels of *PhNF*-*Ys* under several stresses (Figure 7). Based on the RNA-seq data, 9 *NF-Y* genes not detected were omitted from the analysis. The results showed that *PhNF*-*YA6* and -10 and *PhNF-YB13* were regulated by drought stress (Figure 7a). *PhNF-YA6* and *PhNF*-*YA10* reached a peak at 6 h and subsequently decreased to a normal level after 12 h of treatment, while *PhNF-YB13* decreased sharply after 12 h of treatment. With the salt treatment (Figure 7b), the expression levels of four *PhNF-Ys* (*PhNF-YA5*, -6, and -10 and *PhNF*-*YB3*) were regulated by salt. *PhNF-YA5*, -6, and -10 were induced during the early stages, reached the peak at 12 h, and then decreased. The expression level of *PhNF-YB3* reached the top at 1 h and then quickly decreased to a normal level. Under cold stress (Figure 7c), 10 *PhNF-Ys* were upregulated or downregulated at different timepoints. For NF-YA subfamily members, *PhNF-YA1* and *PhNF*-*YA7* were obviously downregulated and *PhNF*-*YA4*, -5, -6, and -10 were markedly upregulated at one or more timepoints, suggesting these genes might have obtained divergent functions under cold stress during evolution. In addition, three *NF*-*YB* (*PhNF*-*YB1*, -3, and 2) members were significantly inhibited after 12 h of treatment. *PhNF*-*YB13* showed a gradual increase from 3 to 12 h. With hot stress (Figure 7d), four genes were induced, and five genes decreased. *PhNF-YA1*, -2, and -10 were increased within 1 h and *PhNF*-*YC1* exhibited a gradual increase. *PhNF-YA4*, -5, and -6 and *PhNF*-*YB13* decreased immediately in 1 h. The expression of *PhNF-YA7* was the lowest at 6 h. These results suggested that the *PhNF*-*Y*s had a faster response to hot stress. Notably, we found that more *NF*-*Y* genes participated in temperature stress response, suggesting that these genes might be key candidates for improving tolerance to temperature stress.

## 3. Discussion

Based on the petunia draft genome and the sequences of *Arabidopsis* NF-Y family members, we initially identified 10 NF-YAs, 13 NF-YBs, 4 NF-YCs, 3 NC2βs, and 1 NC2α, Since NC2α/β could not pair with NF-YB/C [50], these four members were removed from further analyses. For the *PhNF*-*YC* subfamily, only 4 members were found, less than 10 members of the *Arabidopsis NF*-*YC* gene family. The reduced numbers of *PhNF*-*YC* genes could result in a reduction of heterotrimers by two-thirds in theory. Smaller amounts of NF-YC members could also decrease the high redundancy of the NF-Y family in plants.

Multiple alignments showed that most PhNF-Y proteins contained the conserved regions responsible for subunit interaction and DNA binding (Figure 1), which were also found in other plants, mammals, and yeasts [32,38]. Previous studies have shown that the DNA binding domain in the C-terminus of NF-YA could bind to the CCAAT sites [41]. In this study, PhNF-YA4 and PhNF-YA6, which lacked the conserved DNA binding domain, might fail to recognize the CCAAT sites. The functions of these two members were worth further investigation. Additionally, although most required AA were conserved, PhNF-YC4 still exhibited some alterations in the NF-YA interaction domain. In consideration of the absence of homologues of the *PhNF-YC4* gene in *Arabidopsis*, the ability to interact with the NF-YA subunit and the biological function of PhNF-YC4 required further testing.

To explore the functions of these *PhNF*-*Ys*, exon-intron structures and evolutionary analysis were performed (Figure 2 and Figure 3). The number and distribution of introns and exons were similar to previous results [4,8,37], suggesting that the functions of *PhNF-Ys* might be similar to homologues genes in other species. In phylogenetic tree analysis, genes showing a close evolutionary relationship usually have a similar function, so we can predict the functions of *PhNF*-*Ys* based on the known functions of *AtNF-Ys*. For example, *AtNF-YB3* was associated with the development of root tissue and flowering time [51], suggesting that its homologues gene, *PhNF-YB3*, might have similar functions. The higher expression level of *PhNF-YB3* in leaf tissue further supported this possibility. *AtNF-YA1* and *AtNF-YA9* were reported to be critical regulators of embryo development [21,52], suggesting that the *PhNF-YA* genes clustered in the same clade might have similar functions. *PhNF-YC2* and *PhNF-YC4* were distant to *NF-YCs* in *Arabidopsis*, suggesting that these genes might have a unique role in determining specific traits in petunias. Moreover, the corresponding heterotrimeric complexes might be quite different from that in *Arabidopsis*.

Previous studies have shown that miRNA169 could direct the cleavage of the mRNA of *NF-YA* to regulate abiotic stress responses in different plant species [26,47,53]. In our study, we identified eight *PhNF-YA* genes possessing complementary sequences of different miRNA169 members, further supporting the existence of the miRNA169/NF-YA module in petunias. It was noted that some *NF-YA* genes contained the binding sites of other 13 miRNA family, including miR156, 159, 168,171, 172, 395, 482, 6164, 8016, 6149, 171(V), and 172(v). The functions of some miRNA were well studied in plants. For example, miR156 was a key component of the aging pathway through directly regulating its targets SQUAMOSA PROMOTER BINDING PROTEIN-LIKE (SPL) at a posttranscriptional level [45]. miRNA164 is involved in plant growth and abiotic stress responses through guiding the cleavage of the mRNAs of *NAC* genes [54]. The miR172 regulated flowering time and vegetative phase change through clearing the mRNA of *AP2* and *AP2-like* genes [45]. In our study, *PhNF-YA1*, -2, -5, -7, and -10 showed an obvious stress response. *PhNF-YA3* had a higher expression level in flower tissue. The functions of these *PhNF-YA* genes and the underlying relationships of *PhNF-YA* and miRNA were worth further investigation. In addition, *PhNF-YB2*, -8, -10, and -12 contained binding sites of miRNA156. In our previous study, *CmNF-YB8* was shown to regulate flowering time via directly binding to the promoter of *cmo-MIR156* [55]. In this study, the expression level of *PhNF-YB8* was relatively constant. The function of *PhNF-YB8* and the relationship of PhNF-YB8 and miR156 in petunias might differ from the situation in chrysanthemum. For the NF-YC subfamily, we first revealed that *PhNF-YC2* contained target sites of miRNA164 and miRNA171(v), and *PhNF-YC3* contained target sites of miRNA394. Both miR164 and miR171 were involved in stress response [45], and miR394 played roles in flower development [45]. The deeper relationships of NF-YC and miRNAs need to be explored.

Many *NF-Y* genes have been reported to participate in drought and salt stress responses. In previous studies, *NF-Y* genes regulated drought tolerance via different mechanisms, including the ABA-dependent pathway, the ABA-independent pathway, and even novel mechanisms. In our study, only *PhNF-YA6* and -10 and *PhNF-YB13* responded to drought. In previous studies, AtNF-YA6 involved ABA signaling [34], and *GmNF-YA21*, the homologues of *AtNF-YA10*, played roles in drought response [34]. Our results indicated that *PhNF-YA6* and *PhNF-YA10* were highly expressed in root tissue, which had a close relationship with stress tolerance, supporting the idea that *PhNF-YA6* and *PhNF-YA10* might be associated with drought response. *PhNF-YB13* showed distant relationships with other NF-YB members, and had a high expression in leaves, roots, and stems, so its function requires further study. Salt stress showed a similar pattern of drought stress, and *PhNF-YA5*, -6, and -10 were quickly induced and were highly expressed in roots. In *Arabidopsis*, the induction of *AtNF-YA5* under drought occurred at both the transcriptional and posttranscriptional levels [15]. The tissue-specificity of *PhNF-YA5* expression indicated that it had higher expression in roots and stems. In our study, *PhNF-YA5* was weakly induced after 3 h of drought stress. We speculated that *PhNF-YA5* might regulate salt tolerances through regulating root or stem development. It has been well known that the mechanisms of drought response and salt response overlap to some degree. It is easier to understand that *PhNF-YA5*, -6, and -10 responded to both drought and salt. *PhNF-YB3* was quickly induced by salt. *AtNF-YB3,* the homologues of *PhNF-YB3,* was identified to regulate flowering time [34]. In our study, *PhNF-YB3* showed higher expression in leaf and flower tissue. We concluded that *PhNF-YB3* should play roles in flowering time regulation and salt response, which requires further evidence.

There is little evidence that *NF-Y* genes are involved in cold and hot responses [30]. In our study, 10 *PhNF-Y* genes were obviously induced or suppressed by cold responses, and 9 *PhNF-Y* genes quickly responded to hot responses. Among these genes, seven (*PhNF-YA1*, -4, -5, -6, -7, and -10 and *PhNF-YB13*) were involved in both cold and hot responses. The expression of *PhNF-YA4*, -5, and -6 increased under cold responses and decreased under hot responses. The performances of *PhNF-YA7* and -10 and *PhNF-YB13* were similar under hot and cold responses. Moreover, except for *PhNF-YA4*, six other genes showed high expression in roots, indicating the underlying mechanisms that might be overlapped to a certain extent. Notably, we found that *PhNF-YA6* and *PhNF-YA10* responded to all stresses, including drought, salt, and cold and hot stress, indicating their multifunctional roles under different abiotic stresses. In our studies, more *PhNF-Y* genes responded to hot and cold stresses than to drought and salt. We concluded that *PhNF-Y* genes might play unknown, important roles in temperature stress. However, we could not rule out the possibility that different treatment concentrations and times result in dramatic expression changes under drought or salt treatment. In conclusion, the results of the stress response experiments, together with the tissue-specific expression, suggested that many *PhNF-Y* genes might respond to stresses through regulating root or leaf development. These *PhNF-Y* genes deserve further research and might be utilized for improving the abiotic stresses tolerance of petunias.

## 4. Materials and Methods

### 4.1. Identification, Alignments, and Phylogenetic Analysis of NF-Y Members

The sequences of 36 *Arabidopsis* NF-Y proteins were obtained from the Arabidopsis Information Resource (TAIR, https://www.arabidopsis.org/). The full length of all *Arabidopsis* NF-Y proteins were used as queries to blast petunia NF-Y protein sequences from Sol Genomics Network (SGN, https://www. solgenomics.net/). Alignments of full-length amino acid sequences of identified PhNF-Ys and AtNF-Ys were performed using ClustalX (http://www.clustal.org/) and BioEdit. Based on the sequence alignments generated by ClustalX, all potentially redundant NF-Y sequences were discarded. Phylogenetic trees were constructed using MEGA6 [56] and the neighbor-joining method with 1000 bootstrap replicates. The dendrograms were drawn by the online tool of iTOL (https://itol.embl.de/).

Molecular weight (MW) and isoelectric point (pI) of the deduced amino acid sequences were predicted with the Sequences Manipulation Suite (https://www.genscript.com/sms2/index.html).

### 4.2. Conserved Motifs and Gene Structure Analyses

PhNF-Y conserved motifs were identified using the Multiple Em for Motif Elicitation (MEME, http://www.meme.sdsc.edu/meme/meme.html). The parameter settings were as follows: the maximum number of motifs was set to 10, the width range was 6–55, and other factors were at default selections [8].

Information about Genomic DNA Sequences and Coding Sequences (CDS) were obtained from the SGN database. The exon-intron structures of the *PhNF-Ys* were constructed using Gene Structure Display Server 2.0 (http://gsds.cbi.pku.edu.cn/).

### 4.3. miRNA Target Site Prediction

Genomic DNA sequences of 27 AtNF-Ys were used to predict the possible targets of all mature miRNA members using the online tool PsRNA server (http://plantgrn.noble.org/psRNATarget/). Parameters are set as follows: The maximum expectation value is 3.5, the length for complementarity scoring (hsp size) is 21 bp, target accessibility (UPE) is 25, flanking sequence length is 17 bp in upstream and 13 bp in downstream, and the translational inhibition range is 9–11NT.

### 4.4. Plant Material, Tissue, and Stress Treatment

Petunia “*Ultra*” seeds were grown in 7 cm diameter pots containing sterile nutrient soil and cultured in a room at 22 ± 3 °C under an LD cycle (14 h light/10 h dark). The 10-leaf stage seedlings were exposed to varieties of abiotic stresses in chambers, including cold stress (4 °C), hot stress (40 °C), drought stress (20% polyethylene glycol-6000 (PEG-6000)), and salt stress (500 mM NaCl). For the temperature treatment, seedlings were exposed at either 4 °C or 40 °C in a chamber with an LD cycle (14 h light/10 h dark), after which the second fully expanding leaves from the top were sampled. For the NaCl and PEG 6000 assays, seedlings were transferred to 22 °C water containing the stress agent in chambers at 22 °C under an LD cycle (14 h light/10 h dark), and the second fully expanding leaves from the top were sampled [57]. Samples were collected at 0 h, 1 h, 3 h, 6 h, and 12 h, immediately frozen in liquid nitrogen, and stored at −80 °C. Each treatment was replicated three times.

In the tissue expression pattern experiment, samples of roots, stems, and leaves were collected from seedlings without bloom. The first flowers of three individual plants were collected as flower samples. The samples were frozen in liquid nitrogen and stored at −80 °C.

### 4.5. Transcriptome Data Analysis

Total RNA samples were extracted from the above samples using TRIZOL (Invitrogn, Carlsbad, CA, USA) following the manufacturer’s protocol [58]. The integrity of the RNA samples was confirmed by electrophoresis on 1% agarose gels, and the amount and quality of RNA were detected by NanoDrop 2000 Spectrophotometer (Thermo Scientific, Waltham, MA, USA) [37]. RNA-Seq libraries were prepared and sequenced using a HiSeq 2000 (Illumina) at the Shanghai Origingene Biotechnology Co. Ltd. (http://www.origin-gene.com/). RNA-seq data were processed and assembled as previously described [55]. After removing low-quality reads, adapters, and barcode sequences, high-quality clean reads were assembled de novo into contigs. The resulting contigs were blasted against *Petunia axillaris* draft genome sequence databases (https://www.solgenomics.net/organism/ Petunia_axillaris/genome) with a cutoff E value of 1 × 10^−5^. The Reads Per Kilobase of exon model per Million mapped reads (FPKM) values of all genes were determined with a treatment of log_2_(X), and subsequently used for generating heatmaps. Finally, a series of heat maps was generated using TBtools [59].

## Figures and Tables

**Table 1:** Basic information of the Nuclear Factor Y (NF-Y) gene family in *Petunia*.

Gene ID	Gene Name	mRNA Length (bp)	No. of Introns	Peptide Residues (aa)	MW (kDa)	pI
Peaxi162Scf00004g02521	PhNF-YA1	933	4	310	33.91	7.34
Peaxi162Scf00339g00512	PhNF-YA2	957	4	312	34.05	9.85
Peaxi162Scf00038g01819	PhNF-YA3	1065	4	327	36.13	9.85
Peaxi162Scf00267g00038	PhNF-YA4	792	4	286	31.70	9.37
Peaxi162Scf00953g00217	PhNF-YA5	861	5	298	33.54	9.33
Peaxi162Scf00003g04130	PhNF-YA6	585	2	354	39.76	6.94
Peaxi162Scf00498g00617	PhNF-YA7	897	5	318	34.72	10.04
Peaxi162Scf00809g00019	PhNF-YA8	987	4	263	29.51	9.93
Peaxi162Scf00032g00323	PhNF-YA9	939	4	296	33.26	7.47
Peaxi162Scf00211g00239	PhNF-YA10	888	6	194	22.19	10.39
Peaxi162Scf00195g00082	PhNF-YB1	492	3	163	17.54	5.32
Peaxi162Scf00146g01325	PhNF-YB2	420	2	140	15.58	5.29
Peaxi162Scf00047g02011	PhNF-YB3	606	0	201	20.52	6.68
Peaxi162Scf00037g00085	PhNF-YB4	423	0	140	15.90	4.56
Peaxi162Scf00534g00022	PhNF-YB5	420	0	139	15.71	4.89
Peaxi162Scf00827g00055	PhNF-YB6	642	0	213	23.69	5.10
Peaxi162Scf00525g00067	PhNF-YB7	696	0	231	26.03	6.95
Peaxi162Scf00208g00014	PhNF-YB8	534	3	177	19.36	5.73
Peaxi162Scf00139g00134	PhNF-YB9	681	1	226	25.43	6.79
Peaxi162Scf00005g00493	PhNF-YB10	546	4	181	19.68	6.79
Peaxi162Scf00067g00163	PhNF-YB11	501	0	166	18.71	8.06
Peaxi162Scf00399g00108	PhNF-YB12	510	1	169	19.32	6.94
Peaxi162Scf01006g00035	PhNF-YB13	588	0	195	21.66	6.54
Peaxi162Scf00089g00023	PhNF-YC1	732	0	243	26.20	5.03
Peaxi162Scf00134g02029	PhNF-YC2	873	1	291	32.20	6.08
Peaxi162Scf00128g00124	PhNF-YC3	513	0	170	18.04	4.25
Peaxi162Scf00102g01519	PhNF-YC4	327	1	108	11.92	10.25

MW: molecular weight; pI: isoelectric point.

## Supplementary Materials

The following are available online at https://www.mdpi.com/2223-7747/9/3/336/s1. Table S1: Identification of mature miRNAs and targeted *NF-Y* genes in *Petunia*. Table S2: Spectrophotometric analyses of RNA samples from plants under different stress treatments. Table S3: Spectrophotometric analyses of RNA samples from different tissues. Figure S1: Electropherograms of RNA samples.
