# Supplementary material for: Genome-Wide Identification and Expression Profile Analysis of the NF-Y Transcription Factor Gene Family in Petunia hybrida
Source: Plants (Basel). 2020 Mar 6;9(3):336. doi: 10.3390/plants9030336 (PMC7154908; doi:10.3390/plants9030336)
Supplement: Supplementary file 1 [file plants-09-00336-s001.zip › plants-728687-supplementary-for proofreading/plants-728687-supplementary-for proofreading.docx]

Supplementary


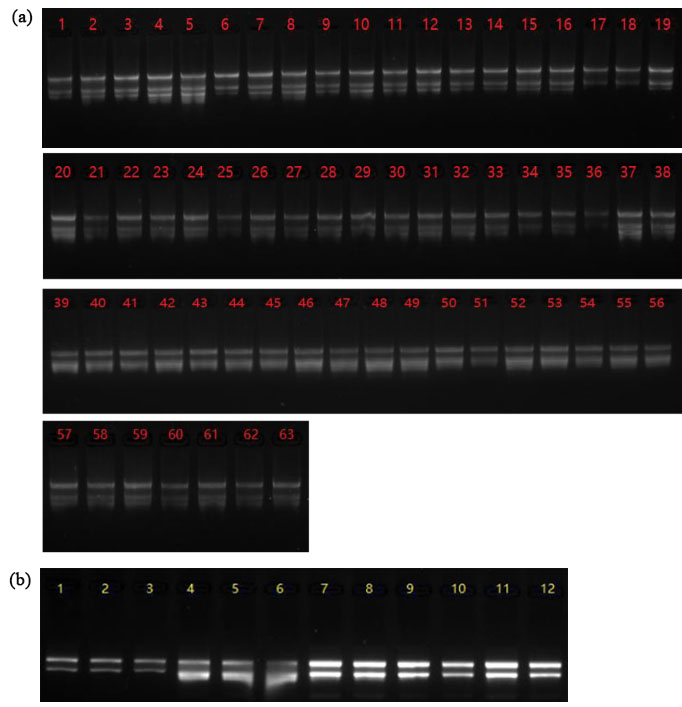


**Figure S1.** Electropherograms of RNA samples. (**a**) Electropherograms of RNA samples from plants under different stress treatments. 1.CK0h-1; 2. CK0h-2; 3. CK0h-3; 4. CK1h-1; 5. CK1h-2; 6. CK1h-3; 7. Salt1h-1; 8. Salt1h-2; 9. Salt1h-3; 10. Deh1h-1; 11. Deh1h-2; 12. Deh1h-3; 13. cold1h-1; 14. cold1h-2; 15. cold1h-3; 16. Hot1h-1; 17. Hot1h-2; 18. Hot1h-3; 19. CK3h-1; 20. CK3h-2; 21. CK3h-3; 22. Salt3h-1; 23. Salt3h-2; 24. Salt3h-3; 25.Deh3h-1; 26. Deh3h-2; 27. Deh3h-3; 28. cold3h-1; 29. cold3h-2; 30. cold3h-3; 31. Hot3h-1; 32. Hot3h-2; 33. Hot3h-3; 34. CK6h-1; 35. CK6h-2; 36. CK6h-3; 37. Salt6h-1; 38. Salt6h-2; 39. Salt6h-3; 40. Deh6h-1; 41. Deh6h-2; 42. Deh6h-3; 43. cold6h-1; 44. cold6h-2; 45. cold6h-3; 46. Hot6h-1; 47. Hot6h-2; 48. Hot6h-3; 49. CK12h-1; 50. CK12h-2; 51. CK12h-3; 52. Salt12h-1; 53. Salt12h-2; 54. Salt12h-3; 55. Deh12h-1; 56. Deh12h-2; 57. Deh12h-3; 58. cold12h-1; 59. cold12h-2; 60. cold12h-3; 61. Hot12h-1; 62. Hot12h-2; 63. Hot12h-3. (**b**) Electropherograms of RNA samples from different tissues.1. flower-1; 2. flower-2; 3. flower-3; 4. leaf-1; 5. leaf-2; 6. leaf-3; 7. stem-1; 8. stem-2; 9. stem-3.

**Table S2.** Spectrophotometric analyses of RNA samples from plants under different stress treatments.

| **Number** | **Sample Name** | **Content (ng/ul)** | **OD 260/280** | **OD 260/230** | **Volume (ul)** | **Total Amount (ug)** |
| --- | --- | --- | --- | --- | --- | --- |
| 1 | CK0h-1 | 373.5 | 2.19 | 2.10 | 45 | 16.8 |
| 2 | CK0h-2 | 503.1 | 2.15 | 2.13 | 45 | 22.6 |
| 3 | CK0h-3 | 499.1 | 2.17 | 2.15 | 45 | 22.5 |
| 4 | CK1h-1 | 771.1 | 2.17 | 2.13 | 45 | 34.7 |
| 5 | CK1h-2 | 728.2 | 2.18 | 2.09 | 45 | 32.8 |
| 6 | CK1h-3 | 463.9 | 2.20 | 2.12 | 45 | 20.9 |
| 7 | Salt1h-1 | 675.4 | 2.18 | 2.11 | 45 | 30.4 |
| 8 | Salt1h-2 | 642.9 | 2.16 | 2.14 | 45 | 28.9 |
| 9 | Salt1h-3 | 470.2 | 2.20 | 2.11 | 45 | 21.2 |
| 10 | Deh1h-1 | 623.8 | 2.17 | 2.12 | 45 | 28.1 |
| 11 | Deh1h-2 | 462.8 | 2.19 | 2.11 | 45 | 20.8 |
| 12 | Deh1h-3 | 570.4 | 2.19 | 2.12 | 45 | 25.7 |
| 13 | cold1h-1 | 461.0 | 2.19 | 2.11 | 45 | 20.7 |
| 14 | cold1h-2 | 510.7 | 2.14 | 2.09 | 45 | 23.0 |
| 15 | cold1h-3 | 702.0 | 2.16 | 2.14 | 45 | 31.6 |
| 16 | Hot1h-1 | 768.1 | 2.19 | 2.07 | 45 | 34.6 |
| 17 | Hot1h-2 | 288.7 | 2.20 | 2.08 | 45 | 13.0 |
| 18 | Hot1h-3 | 533.4 | 2.14 | 2.12 | 45 | 24.0 |
| 19 | CK3h-1 | 735.2 | 2.18 | 2.11 | 45 | 33.1 |
| 20 | CK3h-2 | 752.6 | 2.18 | 2.13 | 45 | 33.9 |
| 21 | CK3h-3 | 421.0 | 2.18 | 2.11 | 45 | 18.9 |
| 22 | Salt3h-1 | 829.1 | 2.17 | 2.13 | 45 | 37.3 |
| 23 | Salt3h-2 | 642.9 | 2.17 | 2.11 | 45 | 28.9 |
| 24 | Salt3h-3 | 643.9 | 2.17 | 2.11 | 45 | 29.0 |
| 25 | Deh3h-1 | 370.3 | 2.19 | 2.11 | 45 | 16.7 |
| 26 | Deh3h-2 | 734.9 | 2.16 | 2.12 | 45 | 33.1 |
| 27 | Deh3h-3 | 438.4 | 2.20 | 2.12 | 45 | 19.7 |
| 28 | cold3h-1 | 674.4 | 2.17 | 2.11 | 45 | 30.3 |
| 29 | cold3h-2 | 560.0 | 2.19 | 1.92 | 45 | 25.2 |
| 30 | cold3h-3 | 692.7 | 2.15 | 2.11 | 45 | 31.2 |
| 31 | Hot3h-1 | 755.2 | 2.16 | 2.11 | 45 | 34.0 |
| 32 | Hot3h-2 | 728.2 | 2.16 | 2.09 | 45 | 32.8 |
| 33 | Hot3h-3 | 556.6 | 2.17 | 2.05 | 45 | 25.0 |
| 34 | CK6h-1 | 406.8 | 2.20 | 2.03 | 45 | 18.3 |
| 35 | CK6h-2 | 398.7 | 2.20 | 2.10 | 45 | 17.9 |
| 36 | CK6h-3 | 255.9 | 2.20 | 1.99 | 45 | 11.5 |
| 37 | Salt6h-1 | 715.0 | 2.18 | 2.11 | 45 | 32.2 |
| 38 | Salt6h-2 | 826.8 | 2.17 | 2.12 | 45 | 37.2 |
| 39 | Salt6h-3 | 595.4 | 2.17 | 2.10 | 45 | 26.8 |
| 40 | Deh6h-1 | 490.5 | 2.17 | 2.11 | 45 | 22.1 |
| 41 | Deh6h-2 | 460.8 | 2.20 | 2.11 | 45 | 20.7 |
| 42 | Deh6h-3 | 605.3 | 2.15 | 2.12 | 45 | 27.2 |
| 43 | cold6h-1 | 415.1 | 2.19 | 2.10 | 45 | 18.7 |
| 44 | cold6h-2 | 466.3 | 2.20 | 2.12 | 45 | 21.0 |
| 45 | cold6h-3 | 501.2 | 2.17 | 2.08 | 45 | 22.6 |
| 46 | Hot6h-1 | 832.5 | 2.17 | 2.12 | 45 | 37.5 |
| 47 | Hot6h-2 | 560.7 | 2.17 | 2.10 | 45 | 25.2 |

**Table S2.** Spectrophotometric analyses of RNA samples from plants under different stress treatments (Continued).

| **Number** | **Sample Name** | **Content (ng/ul)** | **OD 260/280** | **OD 260/230** | **Volume (ul)** | **Total amount (ug)** |
| --- | --- | --- | --- | --- | --- | --- |
| 48 | Hot6h-3 | 804.2 | 2.17 | 2.10 | 45 | 36.2 |
| 49 | CK12h-1 | 702.1 | 2.17 | 2.12 | 45 | 31.6 |
| 50 | CK12h-2 | 494.2 | 2.18 | 2.04 | 45 | 22.2 |
| 51 | CK12h-3 | 251.3 | 2.19 | 2.10 | 45 | 11.3 |
| 52 | Salt12h-1 | 721.5 | 2.16 | 2.12 | 45 | 32.5 |
| 53 | Salt12h-2 | 613.6 | 2.16 | 2.12 | 45 | 27.6 |
| 54 | Salt12h-3 | 358.0 | 2.19 | 2.11 | 45 | 16.1 |
| 55 | Deh12h-1 | 598.8 | 2.16 | 2.09 | 45 | 26.9 |
| 56 | Deh12h-2 | 411.2 | 2.18 | 2.08 | 45 | 18.5 |
| 57 | Deh12h-3 | 730.7 | 2.17 | 2.10 | 45 | 32.9 |
| 58 | cold12h-1 | 977.5 | 2.18 | 2.12 | 45 | 44.0 |
| 59 | cold12h-2 | 370.3 | 2.19 | 2.11 | 45 | 16.7 |
| 60 | cold12h-3 | 734.9 | 2.16 | 2.12 | 45 | 33.1 |
| 61 | Hot12h-1 | 438.4 | 2.20 | 2.12 | 45 | 19.7 |
| 62 | Hot12h-2 | 702.1 | 2.17 | 2.12 | 45 | 31.6 |
| 63 | Hot12h-3 | 494.2 | 2.18 | 2.04 | 45 | 22.2 |

**Table S3.** Spectrophotometric analyses of RNA samples from different tissues.

| **Number** | **Sample Name** | **Content (ng/ul)** | **OD 260/280** | **OD 260/230** | **Volume (ul)** | **Total amount (ug)** |
| --- | --- | --- | --- | --- | --- | --- |
| 1 | flower-1 | 140.3 | 2.19 | 2.05 | 30 | 4.21 |
| 2 | flower-2 | 136.7 | 2.21 | 2.10 | 30 | 4.10 |
| 3 | flower-3 | 113.0 | 2.18 | 2.05 | 30 | 3.39 |
| 4 | leaf-1 | 348.4 | 2.21 | 1.93 | 30 | 10.45 |
| 5 | leaf-2 | 338.2 | 2.18 | 2.13 | 30 | 10.15 |
| 6 | leaf-3 | 300.4 | 2.22 | 1.90 | 30 | 9.01 |
| 7 | stem-1 | 533.2 | 2.19 | 2.10 | 30 | 15.99 |
| 8 | stem-2 | 512.4 | 2.20 | 2.14 | 30 | 15.37 |
| 9 | stem-3 | 433.0 | 2.20 | 2.13 | 30 | 12.99 |
| 10 | root-1 | 278.8 | 2.20 | 2.09 | 30 | 8.36 |
| 11 | root-2 | 507.5 | 2.19 | 2.12 | 30 | 15.22 |
| 12 | Root-3 | 329.7 | 2.21 | 1.93 | 30 | 9.89 |
